# Whole-body insulin resistance leads to accelerated atherosclerosis: role for Nox2 NADPH oxidase

**DOI:** 10.1530/VB-23-0018

**Published:** 2024-11-08

**Authors:** Azhar Maqbool, Hema Viswambharan, Anna Skromna, Natallia Makava, Heba Shawer, Katherine Bridge, Shovkat Kadirovich Muminov, Helen Imrie, Kathryn Griffin, Stephen B Wheatcroft, Piruthivi Sukumar, Richard M Cubbon, Mark T Kearney, Nadira Yusupovna Yuldasheva

**Affiliations:** 1 Leeds Institute for Cardiovascular and Metabolic Medicine, University of Leeds, UK; 2 The Welcome Trust Centre for Human Genetics, University of Oxford, UK; 3 Tashkent Pediatric Medical Institute, Tashkent, Uzbekistan; 4 The Newcastle Upon Tyne Hospitals NHS Foundation Trust, Newcastle Upon Tyne, UK

**Keywords:** atherosclerosis, insulin resistance, Nox2 NADPH oxidase

## Abstract

Insulin resistance underpins the progression of type 2 diabetes mellitus and leads to a collection of risk factors for the development of atherosclerosis. Whether or not insulin resistance at a whole-body level per se leads to accelerated atherosclerosis is unclear. To answer this question, we generated atherosclerosis-prone mice with whole-body insulin resistance secondary to haploinsufficiency of the insulin receptor (IR^+/−^) deficient in ApoE^−/−^ (IR^+/−^/ApoE^−/−^). IR^+/−^/ApoE^−/−^ and ApoE^−/−^ littermates had similar weight, lipids, and glucose tolerance at baseline. After 12 weeks of Western high-cholesterol diet, IR^+/−^/ApoE^−/−^ had significantly more atherosclerosis in the thoracoabdominal aorta and at the level of the aortic sinus than ApoE^−/−^ littermates. Excess Nox2 NADPH oxidase (Nox2) derived superoxide has been suggested to underpin diabetes-related atherosclerosis. In IR^+/−^/ApoE^−/−^ we examined the effect of inhibiting Nox2 using genetic or pharmacological approaches on the development of atherosclerosis. To genetically delete Nox2, we generated IR^+/−/^ApoE^−/−^/Nox2^−/y^ and to inhibit Nox2 pharmacologically, we treated IR^+/−^/ApoE^−/−^ with the peptide Nox2 inhibitor gp91dstat. IR^+/−^/ApoE^−/−^/Nox2^−/y^ had significant disruption of the aortic wall with increased thoracoabdominal atherosclerosis when compared to IR^+/−^/ApoE^−/−^/Nox2^+/y^ littermates. Inhibition of Nox2 using gp91dstat reduced atherosclerosis in the thoracoabdominal aorta of IR^+/−^/ApoE^−/−^. Whole-body insulin resistance accelerates the development of atherosclerosis. Genetic inhibition of Nox2 leads to disruption of the aortic wall in IR^+/−^/ApoE^−/−^ mice with accelerated atherosclerosis, whereas pharmacological Nox2 inhibition reduces atherosclerosis in IR^+/−^/ApoE^−/−^ without disruption of the arterial wall.

## Introduction

Insulin-resistant type 2 diabetes, characterised by loss of normal glucose homeostasis, is a chronic, progressive, and systemic disorder that leads to premature atherosclerosis (1). Several studies have shown that insulin resistance restricted to the endothelial cell (EC) lining of the vasculature leads to accelerated atherosclerosis (2, 3). Whether insulin resistance at a whole-body level before the onset of hyperglycaemia causes accelerated atherosclerosis remains unclear, although long-term epidemiological studies suggest that this may be the case (4, 5). To definitively answer this question in an animal model of human insulin resistance before the onset of dysglycaemia we crossed mice haploinsufficient for the insulin receptor (IR^+/−^) with atherosclerosis-prone Apolipoprotein E holoinsufficient mice (ApoE^−/−^). One pathophysiological process thought to make a major contribution to type 2 diabetes-related atherosclerosis is the unrestrained generation of cytotoxic concentrations of the free radical superoxide from the endothelial lining of the arterial wall (6). This so-called ‘oxidative stress’ has a range of effects that could accelerate the development of atherosclerosis, the principal amongst which is thought to be irreversible oxidative modification of macromolecules, which change the architecture of the arterial wall (7).

We have demonstrated that the principal enzymatic source of superoxide from ECs in insulin resistance is the Nox2 isoform of nicotinamide adenine dinucleotide phosphate oxidase (NADPH oxidase) (8, 9, 10, 11, 12), and inhibition of Nox2 using pharmacological or genetic approaches can reduce superoxide generation, improve vascular function, and, in the case of pharmacological Nox2 inhibition, slow the progression of atherosclerosis in mice with EC-specific insulin resistance (13). A key unanswered question is whether or not insulin resistance at a whole-body level causes atherosclerosis *in vivo*.

Here, we report the following key findings: i) whole-body haploinsufficiency of the insulin receptor in Apolipoprotein E (ApoE) deficient mice leads to accelerated atherosclerosis in the thoracoabdominal aorta and at the level of the aortic sinus. ii) Mice haploinsufficient for the IR deficient in ApoE also deficient in Nox2 (IR^+/−^/ApoE^−/−^/Nox2^−/y^) develop accelerated atherosclerosis and disruption of the aortic wall. iii) Treating IR^+/−^/ApoE^−/−^ mice with the isoform-specific peptidic Nox2 inhibitor gp91dstat retards the development of atherosclerosis without detriment to the architecture of the arterial wall.

## Materials and methods

All experiments were conducted in accordance with accepted standards of humane animal care under the United Kingdom Home Office Project licence no. 40/3523 and P144DD0D6.

### Gene modified mice

To examine the effect of genetic and pharmacological inhibition of Nox2 NADPH oxidase on insulin resistance-related atherosclerosis, we generated two novel gene-modified mice. Mice with whole-body haploinsufficiency of the insulin receptor (IR^+/−^ (14)) were crossed with mice deficient in ApoE (ApoE^−/−^) to generate IR^+/−^/ApoE^−/−^, and then further crossed with Nox2 holoinsufficient mice to generate IR^+/−^/ApoE^−/−^/Nox2^−/y^ (see Supplementary Figure 1 (see section on supplementary materials given at the end of this article) for breeding and genotyping strategy). To determine transgenic status during breeding, mice were genotyped using ear-notch DNA. Three genotyping reactions (ApoE, IR, and Nox2) were established, details of which are provided in Supplementary Figure 2 and in Supplementary Tables 1, 2, and 3. Mice were maintained in a temperature and humidity-controlled environment on a 12 h light: 12 h darkness cycle. Transgenic male mice and their male littermate controls were studied in all experiments.

### Treatment with the Nox2-specific inhibitor gp91dstat

To examine the effect of pharmacological inhibition of Nox2 NADPH oxidase on the development of atherosclerosis in IR^+/−^/ApoE^−/−^ mice, we performed chronic treatment studies using the Nox2-specific inhibitory peptide gp91dstat (13, 15, 16). At 8 weeks of age, mice were placed on a high-fat, high-cholesterol, proatherogenic, Western-style diet (cat. no. 829100, Dietex) for 12 weeks. Four weeks following the commencement of the Western diet, the mice were anaesthetised and osmotic minipumps (Alzet 2004 and 1004) containing 10 mg/kg/day gp91dstat or scrambled peptide were implanted (13). The pumps were replaced after 4 weeks and left in place for a further 4 weeks.

### Metabolic tests

Blood was sampled from the tail vein. Glucose and insulin tolerance tests were performed by blood sampling after an intraperitoneal injection of glucose (1 mg/g; Sigma Aldrich) or human recombinant insulin (0.75 unit/kg: Actrapid; Novo Nordisk), as we described (8, 9, 10, 11, 12). Glucose concentrations were determined in whole blood using a portable metre (Roche Diagnostics). Plasma insulin concentrations were determined by ELISA (Ultrasensitive mouse ELISA; CrystalChem, Downers Grove, IL, USA). Triglycerides and total cholesterol were quantified as we have described previously (11).

### Arterial blood pressure

Systolic blood pressure was measured by tail-cuff plethysmography (Kent Scientific, Torrington, UK) as we have previously described (8, 9, 10).

### Quantification of atherosclerosis

Following 12 weeks on a Western diet, mice were surgically anaesthetised before thorough terminal exsanguination by cardiac puncture followed by cardiac perfusion with PBS at a constant pressure of 100 mm Hg with outflow through the severed inferior vena cava. This was followed by constant pressure perfusion *in situ* with 4% paraformaldehyde. The heart was removed to study the aortic sinus. The thoracic and abdominal aorta were dissected to allow *en face* quantification of plaque (11, 13).

### Histology of aortic sinus

Specimens of the heart were embedded in paraffin or optimal cutting temperature (OCT) compound. Sections were cut at 5 µm for paraffin-embedded and 10 µm for OCT-embedded sections. Sections were cut until the aortic valve cusps were visible for the aortic sinus. Sections were stained with Miller’s elastin/van Gieson (11, 13).

### Elastin fragmentation

Fragmentation of elastin was assessed by counting the number of breaks in the aortic elastin laminae at the level of the aortic sinus in at least five serial sections per animal (13, 17). The number of breaks was expressed per medial area, which was taken to be the area between the internal and external elastic laminae.

### Statistical analysis

The *a priori* selected comparison was to compare triple transgenic mice deficient in Nox2 and their double transgenic littermates with Nox2 intact and gp91dstat-treated mice with their scrambled peptide-treated littermates. Data were analysed using unpaired Student's *t*-tests or Mann–Whitney tests where appropriate using GraphPad Prism 7.05 (*P* < 0.05 was taken as statistically significant, *n* denotes the number of mice per group unless otherwise stated, data expressed as mean ± s.e.m.).

## Results

### Glucose homeostasis, lipids, arterial blood pressure and atherosclerosis in mice with whole-body insulin resistance deficient in Apolipoprotein E (IR^+/−^/ApoE^−/−^)

**Figure 1:**
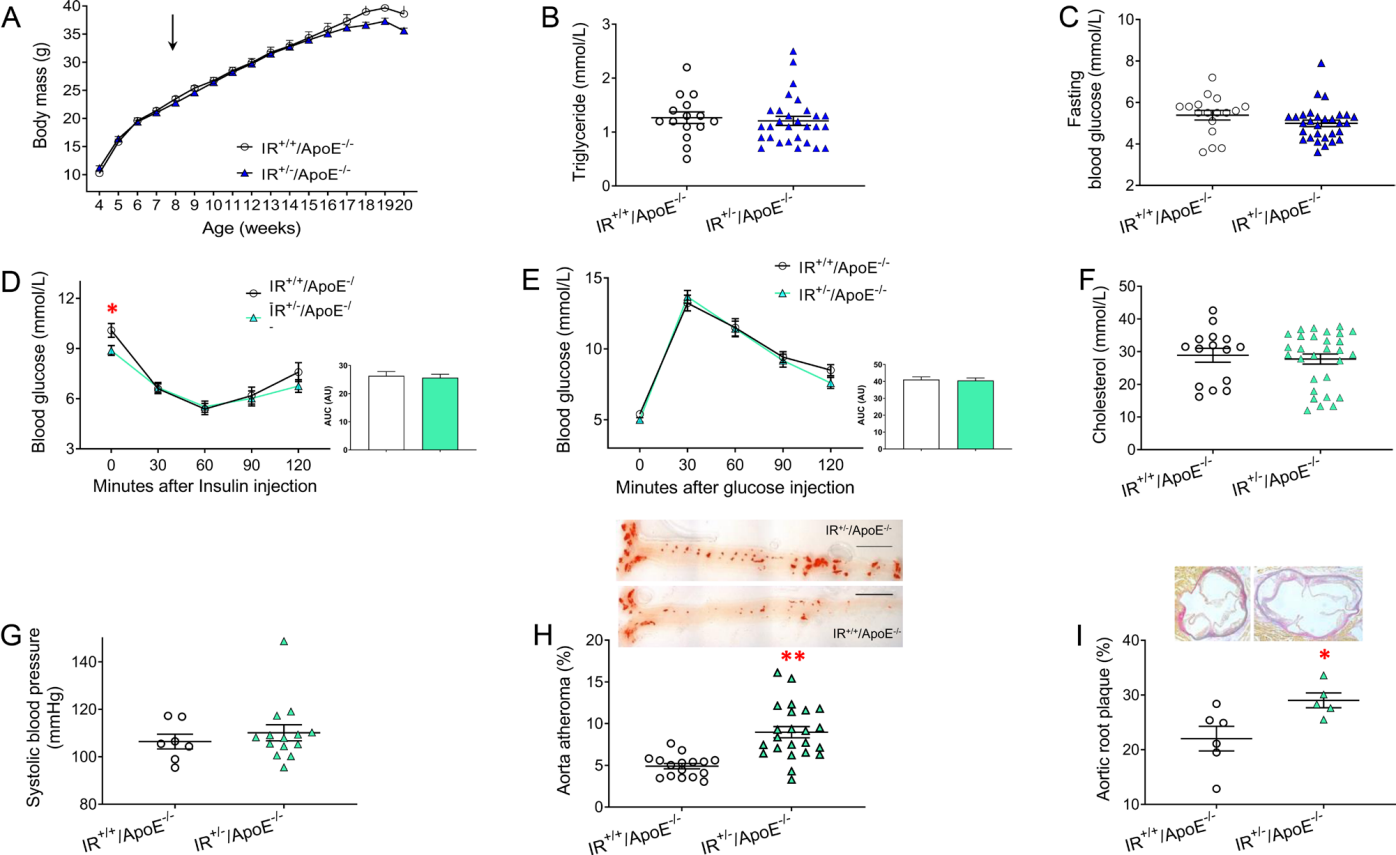
Glucose homeostasis, lipids and atherosclerosis in mice with haploinsufficiency of the insulin receptor deficient in Apolipoprotein E (IR^+/−^/ ApoE^−/−^) fed a high cholesterol Western diet for 12 weeks. (A–G) There was no difference in growth (A, *n* = 18 vs *n* = 26, arrow indicates the commencement of the Western diet), fasting triglycerides (B, *n* = 15 vs. *n* = 29), fasting glucose (C, *n* = 17 vs. *n* = 30), insulin tolerance (D, *n* = 17 vs. *n* = 28), glucose tolerance (E, *n* = 17 vs. *n* = 28), fasting total cholesterol (F, *n* = 15 vs *n* = 29) and systolic blood pressure (G, *n* = 7 vs. *n* = 14) between IR^+/+^/ApoE^−−/^ and IR^+/−^/ApoE^−/−^ mice. (H) IR^+/−^/ApoE^−/−^ mice had significantly more atherosclerosis in the thoracoabdominal aorta than IR^+/+^/ApoE^−/−^ littermates (*n* = 23 vs. *n* = 16). Scale bar = 500 µm. (I) IR^+/−^/ApoE^−/−^ mice had significantly more atherosclerosis at the level of the aortic sinus than IR^+/+^/ApoE^−/−^ mice (*n* = 5 vs. *n* = 6). Data expressed as mean (±s.e.m.), *n* = number of mice per genotype, **denotes *P* < 0.01.

To examine whether insulin resistance at a whole-body level leads to accelerated atherosclerosis, we generated mice with haploinsufficiency of the insulin receptor (IR^+/−^), deficient in Apolipoprotein E (ApoE^−/−^) and fed them a Western diet for 12 weeks. IR^+/−^/ApoE^−/−^ mice had similar weight gain (Fig. 1A), fasting triglycerides (Fig. 1B), fasting blood glucose (Fig. 1C), similar insulin levels (Fig. 1D) and glucose tolerance tests (Fig. 1E) compared to their IR^+/+^/ApoE^−/−^ littermates. In addition, the IR^+/−^/ApoE^−/−^ mice and their IR^+/+^/ApoE^−/−^ littermates had similar fasting cholesterol levels (Fig. 1F) and systolic blood pressure (Fig. 1G). The IR^+/−^/ApoE^−/−^mice, however, developed significantly more atherosclerosis in the thoracoabdominal aorta (Fig. 1H) and aortic sinus (Fig. 1I) compared to their IR^+/+^/ApoE^−/−^ littermates.

### Glucose homeostasis, lipids, arterial blood pressure and atherosclerosis in mice with whole-body insulin resistance deficient in ApoE and Nox2 (IR^+/−^/ApoE^−/−^/Nox2^−/y^)

**Figure 2:**
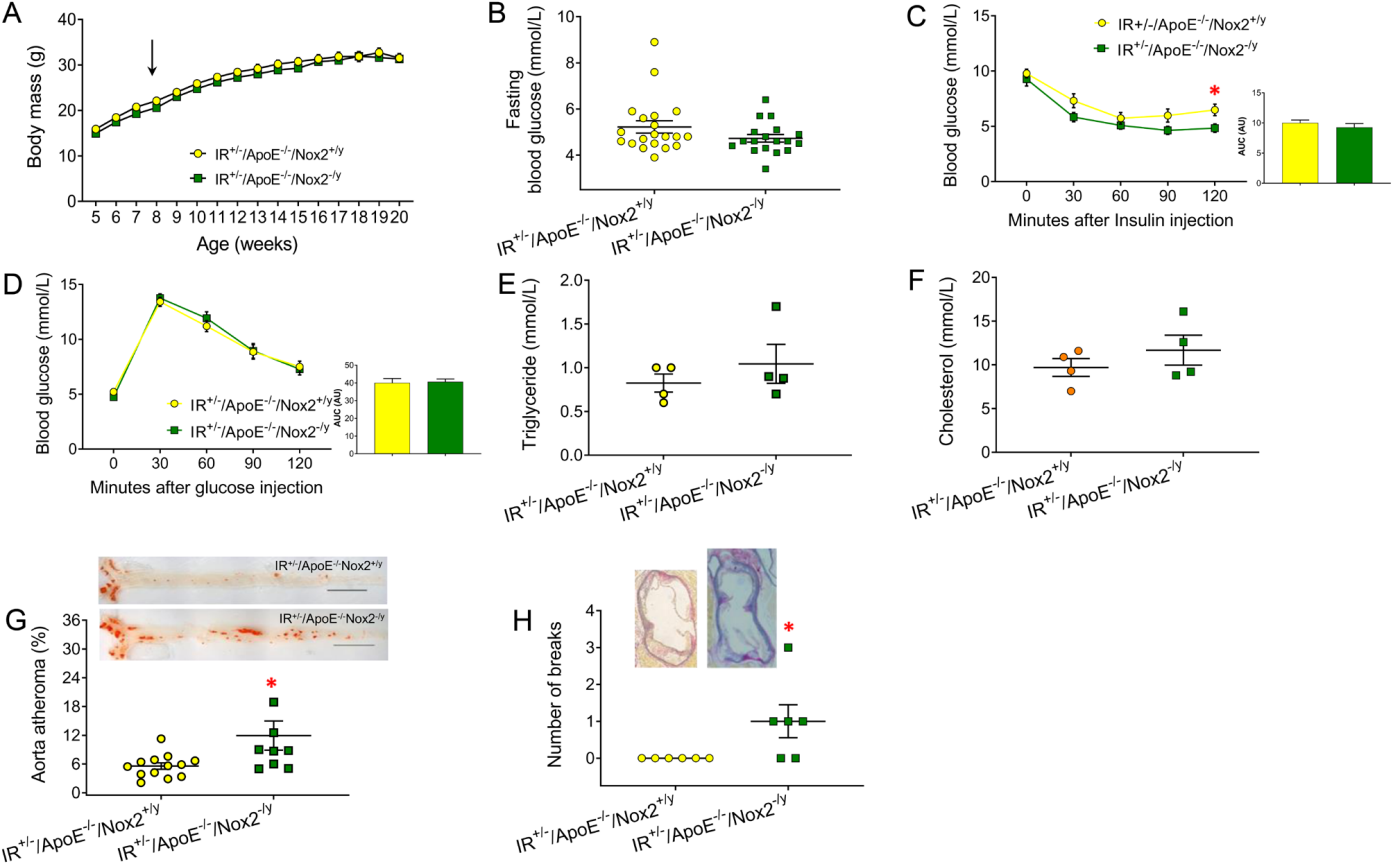
Glucose homeostasis, lipids and atherosclerosis in mice with whole-body haploinsufficiency of the insulin receptor deficient in Apolipoprotein E and Nox2 NADPH oxidase (IR^+/−^/ApoE^−^^/−^/Nox2^−/y^) fed a high cholesterol Western diet for 12 weeks. (A–E) There was no difference in growth (A, *n* = 22 vs. *n* = 21, arrow indicates commencement of the Western diet), fasting blood glucose (B, *n* = 18 vs. *n* = 20), insulin tolerance (C, *n* = 13 vs. *n* = 15), glucose tolerance (D, *n* = 13 vs. *n* = 15), fasting triglyceride (E, *n* = 4 vs *n* = 4) and fasting total cholesterol (F, *n* = 4 vs. *n* = 4) between IR^+/−^/ApoE^−/−^/Nox2^−/y^ and IR^+/−^/ApoE^−/−^/Nox2^+/y^ mice. (G) IR^+/−^/ApoE^−/−^/Nox2^−/y^ had more atherosclerosis in the thoracoabdominal aorta than IR^+/−^/ApoE^−/−^/Nox2^+/y^ mice (*n* = 9 vs. *n* = 13). Scale bar = 500 µm. (H) IR^+/−^/ApoE^−/−^/Nox2^−/y^ mice had increased defects in the aortic wall at the level of the aortic sinus, which was not present in IR^+/+^/ApoE^−/−^/Nox2^+/y^ mice (*n* = 5 vs. *n* = 6). Data expressed as mean (±S.E.M.), *n* = number of mice per genotype, *denotes *P* < 0.05, **P < 0.01.

After 12 weeks on a Western diet, there was no difference in growth (Fig. 2A), fasting glucose (Fig. 2B), insulin (Fig. 2C) and glucose tolerance (Fig. 2D) fasting triglycerides (Fig. 2E), or total fasting cholesterol (Fig. 2F) between IR^+/−^/ApoE^−/−^/Nox2^−/y^ mice and IR^+/−^/ApoE^−/−^/Nox2^+/y^ littermates. However, IR^+/−^/ApoE^−/−^/Nox2^−/y^ mice had more atherosclerosis in the thoracoabdominal aorta compared to IR^+/−^/ApoE^−/−^/Nox2^+/y^ mice (Fig. 2G). There was no difference in the amount of atherosclerosis at the level of the aortic sinus (data not shown), but IR^+/−^/ApoE^−/−^/Nox2^−/y^ mice had evidence of elastin breaks at the level of the aortic sinus, which was less prevalent in IR^+/−^/ApoE^−/−^/Nox2^+/y^ (Fig. 2H).

### The effect of the Nox2 inhibitor gp91dstat on atherosclerotic progression in mice with whole-body insulin resistance deficient in Apolipoprotein E (IR^+/−^/ApoE^−/−^)

**Figure 3:**
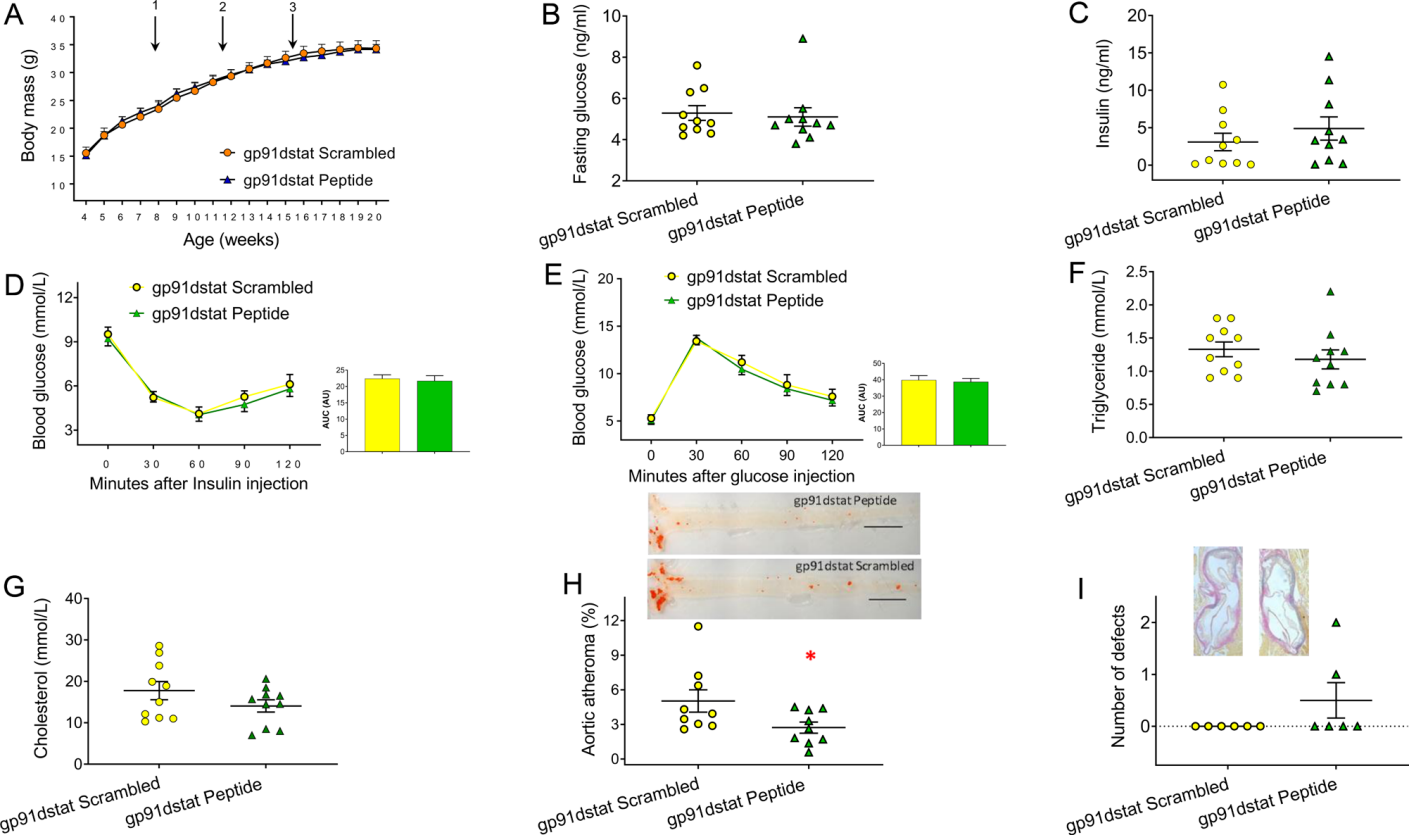
The effect of Nox2-specific inhibitor gp91dstat on glucose homeostasis, lipids and atherosclerosis in mice with whole-body haploinsufficiency of the insulin receptor, deficient in Apolipoprotein E (IR^+/−^/ApoE^−/−^) after 12 weeks on a high-cholesterol Western diet: (A–G) There was no difference in growth (A, arrow 1 denotes commencement of the Western diet, 2 and 3 denote time of minipump implantation), fasting glucose levels (B), random insulin levels (C), insulin tolerance (D), glucose tolerance (E), fasting triglyceride (F) and fasting total cholesterol (G) in IR^+/−^/ApoE^−/−^ mice treated with gp91dstat for 8 weeks compared to those treated with scrambled peptide (all *n* = 10 vs. *n* = 10). (H) IR^+/−^/ApoE^−/−^ mice treated with gp91dstat for 8 weeks showed reduced atherosclerosis in the thoracoabdominal aorta compared to those treated with scrambled peptide (*n* = 9 vs. *n* = 9). Scale bar = 500 µm. (I) There was no difference in the number of defects in the aortic wall at the level of the aortic sinus between IR^+/−^/ApoE^−/−^ mice treated with gp91dstat and those treated with scrambled peptide (*n* = 6 vs. *n* = 6). (Data expressed as mean (±S.E.M.), *n* = number of mice per genotype, *denotes *P* < 0.05).

IR^+/−^/ApoE^−/−^ fed on a Western diet and treated with gp91dstat for 8 weeks showed no difference in growth (Fig. 3A), fasting glucose (Fig. 3B) or random serum insulin concentration (Fig. 3C) compared to those treated with scrambled peptide. Insulin (Fig. 3D) and glucose tolerance (Fig. 3E) were also similar in groups treated with gp91dstat or scrambled peptide, as were fasting triglycerides (Fig. 3F) and total fasting cholesterol (Fig. 3G). However, IR^+/−^/ApoE^−/−^ mice treated with gp91dstat developed less atherosclerosis in the thoracoabdominal aorta than those treated with scrambled peptide (Fig. 3H), although there was no difference in atherosclerosis (data not shown) or elastin breaks at the level of the aortic sinus (Fig. 3I).

## Discussion

Previous studies from our group have shown excess superoxide in mice with whole-body haploinsufficiency of the insulin receptor (8, 10), mice with EC-specific insulin resistance due to expression of a dominant-negative human insulin receptor (9, 10) and mice with excessive insulin signalling in the endothelium, a model of hyperinsulinaemia (11). Studies in humans (18, 19, 20) have implicated NADPH oxidases in obesity and metabolic syndrome-related oxidative stress. In the present report, we demonstrate that mice haploinsufficient for the insulin receptor, and deficient in Apolipoprotein E develop accelerated atherosclerosis, showing for the first time that insulin resistance at a whole body level drives the development of atherosclerosis. Moreover, we demonstrate that genetic deficiency of the superoxide-generating enzyme Nox2 NADPH oxidase in mice haploinsufficient for the insulin receptor, and deficient in Apolipoprotein E leads to accelerated atherosclerosis and significant disruption of the architecture of the arterial wall. In contrast, a potentially more conservative pharmacological approach using a Nox2-specific inhibitor slows the progression of atherosclerosis in mice haploinsufficient for the insulin receptor, and deficient in Apolipoprotein E.

### Atherosclerosis in mice with whole-body insulin resistance

Unlike the present report in studies of mice with haploinsufficiency of the insulin receptor on an Apolipoprotein-deficient background, Rask-Madsen and colleagues (21) did not demonstrate accelerated atherosclerosis using a similar model. There are several potential explanations for this difference; unlike our study, Rask-Madsen *et al.* studied female mice generated as a result of crossing female mice with a null recombination of a single allele of the IR gene (IR^flox/Δ^ ApoE^−/−^). Male breeders had floxed but intact IR genes (IR^flox/flox^ ApoE^−/−^), which were not fully backcrossed onto a C57Bl/6J background. There was a strong tendency for these mice to have enhanced glucose tolerance and thus do not recapitulate the human condition of whole-body insulin resistance previously seen in male IR^+/−^ mice (14, 22). Here, we show that IR haploinsufficient mice deficient in Apolipoprotein have accelerated atherosclerosis in the presence of normal glucose and insulin tolerance tests.

### Complete ablation of Nox2 NADPH oxidase leads to accelerated atherosclerosis and detrimental structural changes to the aorta

We have demonstrated, consistent with our recent findings in mice with endothelium-specific insulin resistance (13), that in the complete absence of Nox2 NADPH oxidase, there is an acceleration of the development of atherosclerosis and significant structural disruption of the aortic wall, with evidence of elastin breaks. The degradation of elastin occurs through a combination of metalloproteinase activity, inflammatory responses triggered by infiltrating immune cells, notably macrophages, oxidative stress, and mechanical forces. As atherosclerosis progresses, calcium and lipid deposition on elastin makes it more prone to proteolytic degradation. This, along with increased elastase activity within atherosclerotic plaque, leads to elastin degradation (23).

Consistent with our data demonstrating an important role for Nox2 in maintaining the integrity of the aortic wall, studies have shown that Nox2 deficiency may accelerate the development of aortic aneurysm (24). On the other hand, using a pharmacological approach to reduce Nox2 activity, the development of atherosclerosis was blunted without inducing disruption of the arterial wall. The present dataset and our previously published work (8, 9, 10, 11, 12, 13) indicate that inhibition, rather than complete ablation of Nox2 may be an attractive treatment target for insulin resistance-related atherosclerosis. The mechanisms underlying the divergent effects of transgenic germline knockdown and pharmacological intervention are likely to be complex and multifactorial. It is noted that in people with chronic granulomatous disease (due to genetic mutations ablating Nox2 activity), loss of Nox2 leads to unresolved chronic inflammation, most readily observed as chronic skin abscesses (25). This is thought to reflect a key role of Nox2-derived superoxide in the clearance of immune-stimulating pathogen-associated and damage-associated molecular patterns during the process of phagocytosis. A similar process may be taking place in the aortic wall of atherosclerosis-prone mice lacking Nox2, leading to the accumulation of immune-stimulating damage-associated molecular patterns that routinely form in plaques. However, it remains possible that Nox2-derived superoxide can also be proatherogenic above a certain threshold, and hence pharmacological inhibition with Gp91-dstat can reduce plaque volume, even though Nox2 deletion induces the converse.

Finally, although it is reported that IR haploinsufficiency in ECs drives plaque formation (2, 3), it is likely that deficiency of IR in multiple cell types contributes to an increase in atherosclerosis. Our data cannot define cell-type-specific contributions or be directly compared to published data with cell lineage-specific (e.g., EC) deletion of IR. However, published data suggest that myeloid IR deletion increases atherosclerosis (26), suggesting that our findings are relevant beyond ECs.

### Limitations

It is important to note some limitations of our work. First, we did not study plaque composition, and it is important to note from a clinical standpoint that small plaques can be vulnerable to rupture, leading to the most important sequelae of atherosclerosis. Second, only male mice were used in this study, as it is established that oestrogen reduces atherosclerotic lesion development in apolipoprotein E-deficient mice. This allowed us to reduce the number of mice studied, though consequently, our findings cannot be extrapolated to female mice. Third, we did not quantify protein-level changes in IR and Nox2 to complement our genotyping data since we have previously published such data from these mice (10, 13, 22, 27).

## Conclusion

Here we present compelling evidence that insulin resistance at a whole-body level leads to accelerated atherosclerosis and that the complete deletion of Nox2 NADPH oxidase further exacerbates the development of insulin resistance-related atherosclerosis. In contrast, we demonstrate that the inhibition of Nox2 using a specific inhibitor slows the development of aggressive atherosclerosis, which is a hallmark of insulin-resistant type 2 diabetes.

## Supplementary Materials

Supplementary Material

## Declaration of interest

The authors declare that there is no conflict of interest that could be perceived as prejudicing the impartiality of the study reported.

## Funding

MTK is the British Heart Foundationhttp://dx.doi.org/10.13039/501100000274 chair of Cardiovascular and Diabetes Research. AS, NM and HV are funded by the British Heart Foundationhttp://dx.doi.org/10.13039/501100000274 Grant PG/14/54/30939. SBW is a BHF Professor of Cardiovascular and Diabetes Research. KJG is an NIHR-funded clinical lecturer.

## Author contribution statement

Conceptualisation: NYY, MTK and RC; Methodology: HV, AS, NM, HMS, KB, HI, KJG, PS and AM; Formal analysis: NYY and MTK; Resources: MTK and NYY; Writing original draft preparation: MTK and NYY; Writing review and editing: MTK, NYY, AM, RMC, SBW and SKM; Supervision: NYY; Funding acquisition: MTK, NYY. All authors have read and agreed to the published version of the manuscript.
