# Supplementary material for: Whole-body insulin resistance leads to accelerated atherosclerosis: role for Nox2 NADPH oxidase
Source: Vasc Biol. 2024 Nov 8;6(1):e230018. doi: 10.1530/VB-23-0018 (PMC11623257; doi:10.1530/VB-23-0018)
Supplement: Supplementary Material [file supplementary_material.pdf]

Whole body insulin resistance leads to accelerated atherosclerosis: role for Nox2 NADPH oxidase.

SUPPLEMENTARY MATERIAL

Maqbool *et al.* 2024

SUPPLEMENTARY FIGURE 1: Breeding and Genotyping Strategy

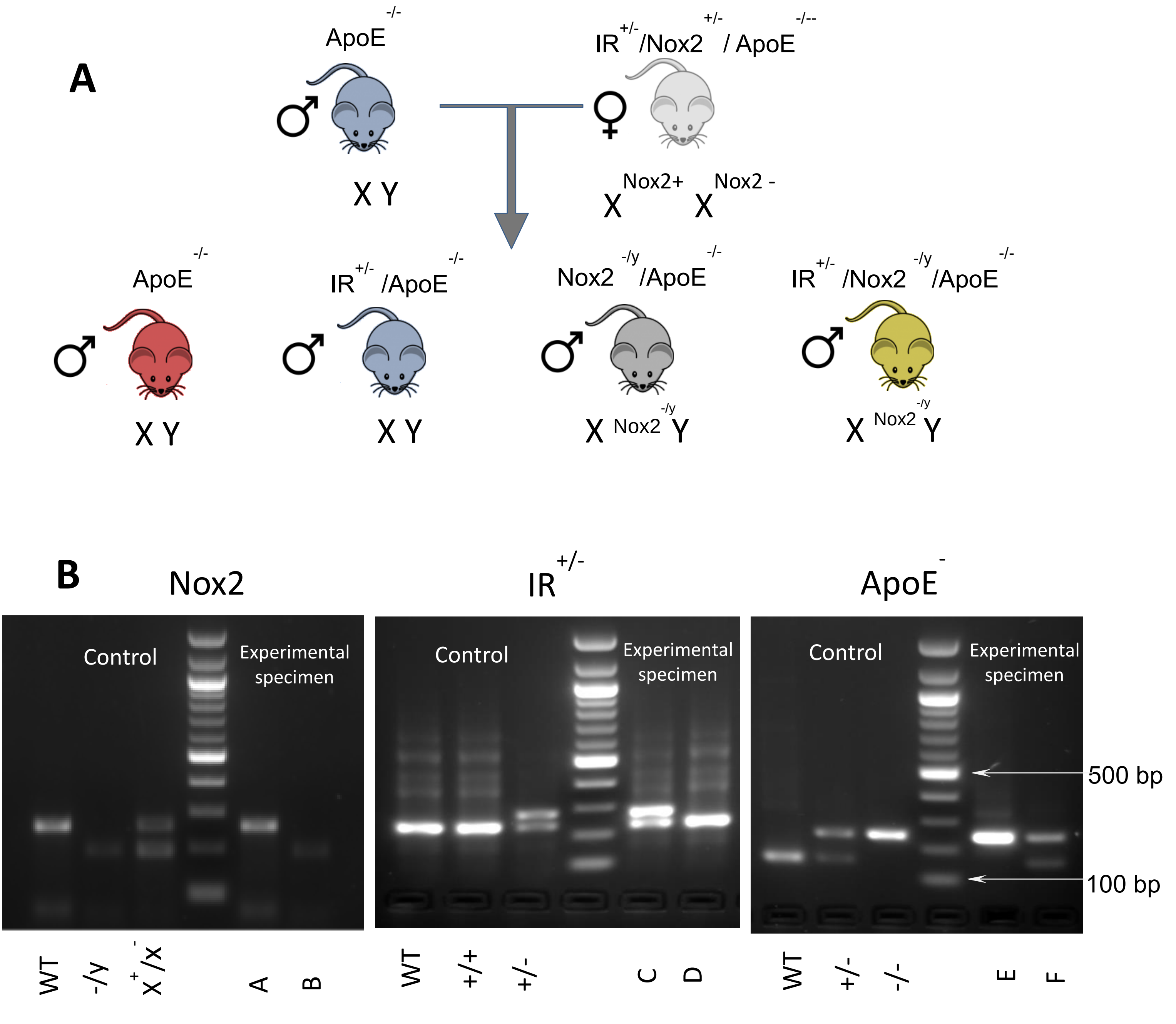

A: Observed male progeny from male ApoE$^{-/-}$ after mating with female ApoE$^{-/-}$/IR$^{+/-}$ /Nox2$^{+/-}$
B: Genotype analysis of PCR products from progeny compared to established control animals: A- Nox2$^{+/y}$; B- Nox2$^{-/y}$; C- IR$^{+/-}$; D- IR$^{+/+}$; E- ApoE$^{-/-}$; F- ApoE$^{+/-}$

SUPPLEMENTARY FIGURE 2: Genotyping Protocol

For ApoE:

0.5µl 10µM Common Primer: 5'-GCCTAGCCGAGGGAGAGCCG-3'

0.5µl 10µM Wild Type Reverse Primer: 5'-TGTGACTTGGGAGCTCTGCAGC-3'

0.5µl 10µM Mutant Reverse Primer: 5'-GCCGCCCCGACTGCATCT-3'

10µl x2 Bio mix red PCR MasterMix (Bioline BIO25006), 12.5µl water and 1µl extracted DNA. PCR cycle conditions are provided in Table S1. Cycles were run using a Verti 96 well thermo cycler (Applied Biosystems). PCR products were then run on a 1.5% agarose gel for 1 hr at 100 V, with a 100 bp ladder (New England Biolabs N0467S). Expected products sizes; were ApoE $^{+/+}$ 155 bp, ApoE $^{-/-}$245 bp, and ApoE $^{-/+}$ 155 & 245 bp.

For IR:

0.5µl 10µM Common Primer 1 5'-AGC TGT GCA CTT CCC TGC TCAC-3'

0.5µl 10µM Wild Type Reverse Primer 2 5'-TTA AGG GCC AGC TCA TTC CTCC-3'

0.5µl 10µM Mutant Reverse Primer 3 5'-TCT TTG CCT GTG CTC CAC TCT CA-3'

10µl x2 Bio mix red PCR MasterMix (Bioline BIO25006), 12.5µl water and 1µl extracted DNA. PCR cycle conditions are provided in Table 4. Cycles were run using a Verti 96 well thermo cycler (Applied Biosystems). PCR products were then run on a 1.5% agarose gel for 1 hr at 100 V, with a 100 bp ladder (New England Biolabs N0467S). Expected products sizes; were WT - 232 bp and IR$^{+/-}$ - 232 bp and 280 bp

For Nox2:

0.5µl 10µM Common Primer 1: 5'-AAGAGAAACTCCTCTGCTGTG AA -3"

0.5µl 10µM Wild Type Reverse Primer 2: 5'-CGCACTGGAACCCCTGAGAAAGG -3"

1µl 10µM Mutant Reverse Primer 3: 5'-GTTCTA ATTCCATCAGAAGCTTAT CG -3'

10μl x2 Bio mix red PCR MasterMix (Bioline BIO25006), 12μl water and 1μl extracted DNA. PCR cycle conditions are provided in Table S3. Cycles were run using a Verti 96 well thermo cycler (Applied Biosystems). PCR products were then run on a 1.5% agarose gel for 1 hr at 100 V, with a 100 bp ladder (New England Biolabs N0467S). Expected products sizes; were Nox2 $^{+/+}$ 240 bp, Nox2 $^{-/-}$195 bp, and Nox2 $^{-/+}$ 195 & 240 bp.

SUPPLEMENTARY TABLE 1: APOE PCR Protocol

PCR	Temperature (°C)	Time	Cycles
Initial denaturation	94	3 min	1
Denaturation	94	20 sec	
Annealing	68	40 sec	35
Extension	72	2 min	

SUPPLEMENTARY TABLE 2: IR PCR Protocol

PCR	Temperature (°C)	Time	Cycles
Initial denaturation	94	1 min	1
Denaturation	94	20 sec	
Annealing	62	20 sec	35
Extension	72	15 sec	
Final Extension	72	4 min	1

SUPPLEMENTARY TABLE 3: Nox2 PCR Protocol

PCR	Temperature (°C)	Time	Cycles
Initial denaturation	94	5 min	1
Denaturation	94	30 sec	
Annealing	55	30 sec	35
Extension	72	30 sec	
Final Extension	72	7 min	1
